# Multi-tissue observation of the long non-coding RNA effects on sexually biased gene expression in cattle

**DOI:** 10.5713/ajas.18.0516

**Published:** 2018-11-28

**Authors:** Joon Yoon, Heebal Kim

**Affiliations:** 1 Department of Natural Science, Interdisciplinary Program in Bioinformatics, Seoul National University, Seoul 08826, Korea; 2 Department of Agricultural Biotechnology, Animal Biotechnology and Research Institute of Agriculture and Life Sciences, Seoul National University, Seoul 08826, Korea

**Keywords:** Cattle, Sexually Dimorphic, Genic lncRNA, RNA-seq, Expression Profile, Tissue Specificity

## Abstract

**Objective:**

Recent studies have implied that gene expression has high tissue-specificity, and therefore it is essential to investigate gene expression in a variety of tissues when performing the transcriptomic analysis. In addition, the gradual increase of long non-coding RNA (lncRNA) annotation database has increased the importance and proportion of mapped reads accordingly.

**Methods:**

We employed simple statistical models to detect the sexually biased/dimorphic genes and their conjugate lncRNAs in 40 RNA-seq samples across two factors: sex and tissue. We employed two quantification pipeline: mRNA annotation only and mRNA+lncRNA annotation.

**Results:**

As a result, the tissue-specific sexually dimorphic genes are affected by the addition of lncRNA annotation at a non-negligible level. In addition, many lncRNAs are expressed in a more tissue-specific fashion and with greater variation between tissues compared to protein-coding genes. Due to the genic region lncRNAs, the differentially expressed gene list changes, which results in certain sexually biased genes to become ambiguous across the tissues.

**Conclusion:**

In a past study, it has been reported that tissue-specific patterns can be seen throughout the differentially expressed genes between sexes in cattle. Using the same dataset, this study used a more recent reference, and the addition of conjugate lncRNA information, which revealed alterations of differentially expressed gene lists that result in an apparent distinction in the downstream analysis and interpretation. We firmly believe such misquantification of genic lncRNAs can be vital in both future and past studies.

## INTRODUCTION

Recent studies have shown the importance of long non-coding RNA (lncRNA) and their annotation is growing with time. The quantified expression levels of lncRNA are at a fair amount and therefore it requires additional consideration if a study deals with the transcriptome. In recent literature [1], the simple definition of a lncRNA is written as non-coding RNAs that are long, stable, commonly spliced, and polyadenylated, plus transcribed from their own promoters. Also, in Zhang et al [2], the author states that most large intergenic non-coding RNAs appear indistinguishable from mRNAs, due to the 5′ cap structures and 3′ poly(A) tails. Such characteristics should arouse a question in a scientist’s mind if the mRNA expression is really from a mRNA. Furthermore, we have to be sure that our mRNA expression level is trustworthy, in order to detect differential gene expression. By adding the lncRNA annotation and accounting for their counts, one can remove ambiguous reads that were originally quantified as mRNA in traditional approaches.

Our understanding of the molecular mechanisms underlying sexual dimorphism remains imperfect. A common practice in transcriptomic analysis is to confirm the gene expression between groups of populations and treatments. While many of dimorphism studies are published for humans, rodents, and fruit flies, only a few were attempted in cattle [3,4]. To add, most bovine transcriptomic research focuses on the tissue growth and development, which are exclusive studies on pre-implantation embryos. Other tissues should also be taken into account for further insight into these mechanisms.

Bovine meat and milk are everyday sources of human nutrition [5]. The gender and gender-specific genes of the food-producing animal are known to affect the quality and quantity of those nutrients [6,7]. Investigation of sexual dimorphism in metabolic tissues such as muscle, liver and adipose tissue from cattle is important for both research and the food production industry. Previous works have already proven the gender- and tissue-specific effects in expression profiles [8]. The between-gender expression should be compared in every tissue that is available.

While the entire brain is filled with sexually dimorphic (or biased) features, the hypothalamus-pituitary axis is a primary structure that controls sexual dimorphism in the central nervous system, as well as peripheral tissues. The pituitary gland regulates the central endocrine system regarding metabolism, sexual maturation, and growth. Its unique cell types in the anterior secrete polypeptide hormones such as growth hormone and gonadotropins, a family of protein hormones including luteinizing hormones and follicle stimulating hormones, by appropriately orchestrating signals from environmental and internal stimuli. Furthermore, profound sex differences exist in hormonal regulation and responses of the pituitary gland to external stressors, which leads to the females displaying a higher vulnerability to various neuropsychiatric disorders [9, 10]. Hence, examination of sexually dimorphic gene expression profiles in the pituitary gland in multiple metabolic tissues will improve our understanding of sexual dimorphism in both metabolic and physiological perspectives [11,12].

In the present study, we aimed to revisit the tissue-specific sexually dimorphic genes that contribute to bovine sexual dimorphism. Generalized linear model is utilized for analyzing complex RNA-seq data from samples collected from several different tissues- liver, muscle, visceral adipose tissue and pituitary gland: a simple two-group comparison for detecting sexually dimorphic genes in each tissue. We followed two pipelines—mRNA annotation only and mRNA with lncRNA annotation—and we report the differences in these approaches for identification of sexually dimorphic genes in several tissues. While a few of the recent studies published results of mRNA and lncRNA profiling in RNA-seq, none revisited old datasets and determined how the list of differentially expressed genes (DEGs) differentiates from the original manuscripts [13].

## MATERIALS AND METHODS

### RNA-seq data processing

The dataset is publicly available from previous literature [13]. However, in their preprocessing of the dataset, the outdated protocol such as reference file, annotation file, alignment and quantification programs have been updated with a more concurrent in-house pipeline. As a note, the sex-chromosome annotation is discarded due to its absence in bosTau8 (the most concurrent bovine reference file). The detailed Animal handling and RNA-seq procedures are provided in the Supplementary File 1 as an excerpt from their original manuscript, as no extra handling or processing of animals and RNA samples were done.

Here, the mRNA pipeline (original) only considers mRNA-to-gene annotation, while our pipeline includes the lncRNA annotation additional to that of the mRNA in the quantification step. After preprocessing of the data, Hisat2 [14] and featureCounts [15] (non-stranded) have been employed to acquire the counts of our features. Hisat2 and featureCounts are a pair of the most concurrent genome-based quantification tools that are compatible with those from the previous study (since validations will be cited). In the process, the general transfer format (GTF) file from University of California, Santa Cruz (UCSC) genome browser has been used, and the lncRNA annotation GTF from ALDB (a domestic-animal long noncoding RNA database) [16] has been utilized. To account for the lncRNAs, we merged the two GTFs as one and quantified with featureCounts. As a result, we achieved 19,212, 19,823, 19,144, 19,918, and 19,208, 19,817, 19,139, 19,913 genes respectively in the 4 tissues (liver, fat, muscle, and pituitary gland) for mRNA and mRNA+lncRNA pipelines. Default options were used except for the unstranded option for both programs. There were a few contaminated and ambiguous samples that had low mapping and annotation rates; such samples are highlighted in the (Supplementary Table 1), and are removed from the analysis for quality control purposes.

### Statistical model for detecting sexually dimorphic genes in each tissue using generalized linear model implemented in edgeR

In a previous study, various sexually dimorphic genes were detected by two group tests in four tissues using the same data [13]. The study considered a count-type distribution such as Poisson and negative binomial (one solution for over-dispersion in Poisson assumption), which is suitable for measuring gene expression from RNA-Seq data given data characteristics. Hence, generalized linear model (GLM) is employed for analysis of RNA-seq data by assuming gene expression as a negative binomial in edgeR to detect DEGs. However, the study used an outdated source, reference (bosTau7 and annotation), while we have updated those sources (bosTau8 and annotation) and added lncRNA information in our protocol.

We chose the simpler model for identification of sexually dimorphic genes using RNA-seq data composed of two factors (sex and tissues). The simplest approach for the analysis of this data is to perform a two-group test between data from female and males in each tissue, separately, as has been performed in previous studies using microarray data. To extend this method in RNA-seq analysis, we employed a GLM with only one explanatory variable (sex group variable) as shown.

log(E(Y))=μ+Sex

The reason behind this is that the tissue specificity is obvious when using the multidimensionality scaling to cluster our expression profiles which shows much higher between-tissue variance than between-individual or –group, as shown in (Supplementary Figure 1). In other words, the tissue has more effect on the expression level than sex or individual, which is concordant with the previous work [13]. When a full model considering the sex and tissue is fitted, the interaction term is significant for a major part of the considered genes; this also means that the sexually dimorphic genes should be separately attested for each tissue. In other words, a simple analysis of deviance (ANODEV) model is more adequate compared to a reduced two-factor model, which includes tissue as a covariate, when the interaction term between sex and tissue is significant, and that effect of sex on gene expression changes with the tissue. Since lncRNA expression is also known for its tissue-specificity [17], we carried out the analysis using the tissue-specific simple ANODEV model. The ANODEV model is a GLM that is frequently used in RNA-seq studies by considering the dependent variable as a poisson distribution; it is a GLM compatible version of analysis of variance analysis [18]. We used a false discovery rate <0.05 significance cutoff for multiple testing adjustments and summarized the significant genes for each tissue separately.

## RESULTS

### Identification of sexually dimorphic genes using the one-way model in each tissue

By using our simple ANODEV model, we identified differentially expressed genes for each pipeline and summarized their results by finding the intersecting genes (concordant genes) and pipeline-specific genes. In the original pipeline, mRNA only annotation, there were 37, 23, 40, and 31 genes significant for muscle, adipose tissue, pituitary gland, and liver, respectively. For our pipeline, with mRNA and lncRNA annotation, 38, 23, 40, and 31 genes were respectively detected. Although the numbers may seem similar, as in pituitary gland, the proportion of intersecting genes is not significant when considering all tissues. Specifically, out of the 23 significant genes, only 3 genes are significant in adipose tissue expression. Other tissues also display a sufficient number of pipeline-specific DEGs as shown in (Table 1).

One can easily observe the different composition of DEGs that results from the addition of lncRNA annotation and their tissues. In the (Additional Figures 1–8) of the supplementary, the top 10 genes of each analysis are illustrated with between-sex boxplots; different gene and rank composition can be seen from the figures, yet, we focus on the downstream analyses of the full DEG lists.

### Identification of sexually dimorphic genes in relation to sex biasedness

Sex biasedness is usually defined by the over-expression in one or the other sex. Hence, the upregulated genes in the female samples can be defined as female-biased, and the genes down-regulated in the female samples can be defined as male-biased in our analysis (the males are used as controls). In the following plot, the first quadrant contains genes that are female-biased in both protocols, the third quadrant contains those biased for males, and the second and fourth quadrants contain ambiguous genes that change regulation direction by the inclusion of lncRNA annotation.

**Figure 1:**
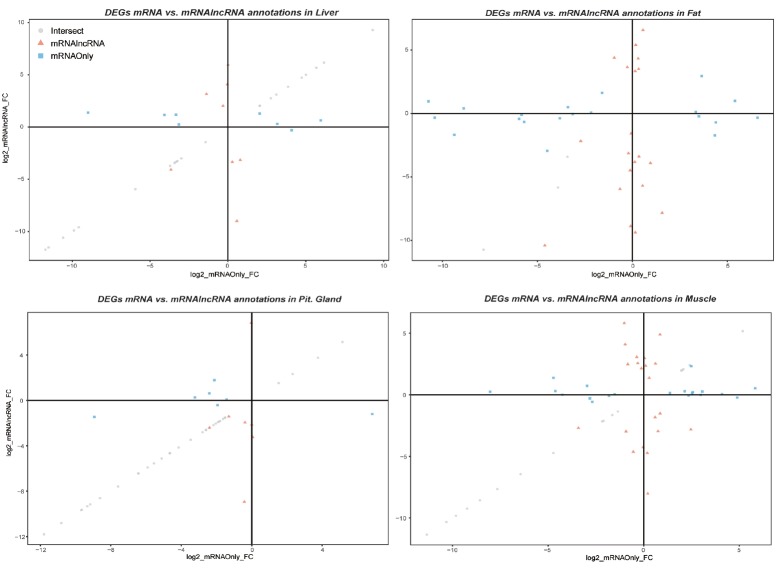
FC-FC plot of 4 tissues. The fold-change in the mRNA pipeline is in the x-axis and fold-change in the suggested pipeline is in the y-axis. The plot displays the difference in fold-changes between the same BAM files under different quantification pipeline. The intersecting genes (circle), and pipeline-specific genes (squares and triangles) are respectively shaped.

Figure 1, is a union of the significant results between the two pipelines, for each tissue; in other words, the figure displays the DEGs from mRNA pipeline and mRNA+lncRNA pipeline combined. The grey circle nodes are the intersection genes, DEGs from the mRNA only pipeline are color-coded with squares, while the DEGs from our pipeline (mRNA & lncRNA) are coded in triangles. One can observe the intersection genes have similar fold-changes, and the pipeline-specific genes have significant differences between the fold-change of expression.

Therefore, we provide separate lists of DEG for the concordant (intersect) and discordant (mRNA only- or mRNA and lncRNA-specific) genes for the different tissues. The circle-shaped genes, which are concordantly significant between the two pipelines, are the most stable and unaffected by the inclusion of lncRNA. We suggest these genes to be carried on to technical validation, for their consistency. Genes such as cytochrome P450 family 7 subfamily A member 1b and epiphycan are included in the intersect list and have been validated with quantitative real-time polymerase chain reaction (qRT-PCR) in the previous study [13] to be sexually dimorphic in both cattle and rat species. The full list of concordant genes is provided in the (Supplementary Table 2). Of course, a comprehensive validation is more appropriate, yet a partial validation of a list implies sex biasedness on the rest of the genes in the list when further technical validation is impossible. To add, the newly detected DEGs in the second pipeline illustrates that checking the DEG list in common practice with our suggested pipeline is vital.

### Downstream gene ontology term and Kyoto encyclopedia of genes and genomes pathway analysis

We analyzed our DEG sets with DAVID [19] for gene ontology (GO) term and Kyoto encyclopedia of genes and genomes (KEGG) pathway analysis. Newly appearing and disappearing list of GO terms and KEGG pathways are identified in (Tables 2, 3), respectively.

**Figure 2:**
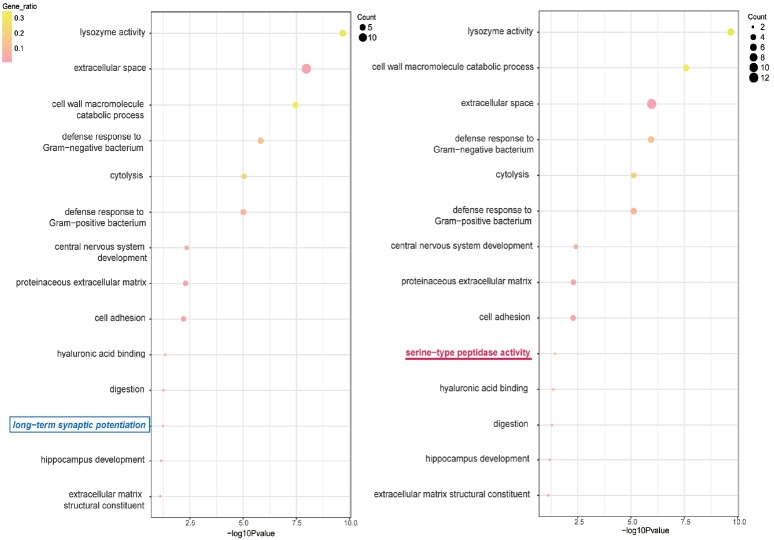
David GO plot of rank and significance in the pituitary gland. Summarized GO terms are illustrated as points with different size for gene count and color for the ratio of gene over total input gene. The corresponding values can be found in (Table 2). The terms are ranked with their corresponding p-values. mRNA-only pipeline on the left and mRNA+lncRNA pipeline on the right. The mRNA-only pipeline-specific term is bolded and boxed, while the mRNA+lncRNA pipeline-specific term is bolded and underlined. GO, gene ontology; lncRNA, long non-coding RNA.

According to Tables 2 and 3, for the muscle, pancreatic secretion pathway (bta04972) and biological process (BP) term of anterior/posterior pattern specification (BP_GO:0009952) are uniquely found for the mRNA only pipeline. In contrast, cholesterol metabolic process (BP_GO:0008203) and chromosome segregation (BP_GO:0007059) is found in our new pipeline. In adipose tissue, motor activity (MF_GO:0003774) and cytoplasm (CC_GO:0005737) are respectively found unique for mRNA only and our pipeline. As for the liver, retinol metabolism (bta00830) and chemical carcinogenesis (bta05204) disappears from the list as the lncRNA is added to the gene list. As bolded and italicized in Table 3, an interesting term that has been removed by the addition of lncRNA in the pituitary gland is the ‘nicotine addiction’. Those genes were mostly down-regulated in the female samples in comparison to males. Additionally, long-term synaptic potentiation (BP_GO:0060291) is found unique for the original pipeline, and serine-type peptidase activity (MF_GO0008236) is found unique for the new pipeline, as shown in (Figure 2). The serine-type peptidase activity has been reported as a sex-biased gene in previous literature [20].

## DISCUSSION

A noteworthy characteristic of some lncRNA is the polyadenylated feature, which should be taken into consideration in RNA-seq studies. In most RNA-seq practices the pipeline, the machines regard all RNAs with poly(A) tails as a mRNA; this may cause some problems if lncRNAs are not filtered out. By performing the commonly followed resequencing-based RNA-seq analysis, we evaluated our dataset and summarized the sexually dimorphic genes in the autosomal regions. Next, we integrated the annotation file with the lncRNA annotations, which will ultimately add a ‘genelist’ to count for in the quantification process. As the (Supplementary Table 1) indicates, the annotated rate = (assigned reads)/(all reads), and ‘Unassigned_Ambiguity’ are slightly greater in the latter protocol. But at the same time, the ‘Unassigned_NoFeatures’ section decreases. This strongly indicates there are lncRNAs that are quantified and is due to the fact that there are more features to assign the reads to; however, the final comparison between the two pipelines should only include the mRNAs that have non-zero counts. The so-called mRNA dataset includes lncRNAs that are mistaken and misquantified as mRNAs. Such lncRNAs can be considered ‘genic lncRNA’ as suggested in the previous literature [21] where lncRNA and mRNA share genomic position in the genic region (Supplementary Figure 2). Such overlapping loci will result ‘Unassigned_Ambiguity” counts by featureCounts, since the program cannot decide which feature to assign the read to. This will result a decrease in the total counts, which ultimately results a decrease in the total number of genes with non-zero counts. If a gene count is zero for all considered samples, number of test decreases, which leads to a less stringent threshold for multiple testing correction.

**Figure 3:**
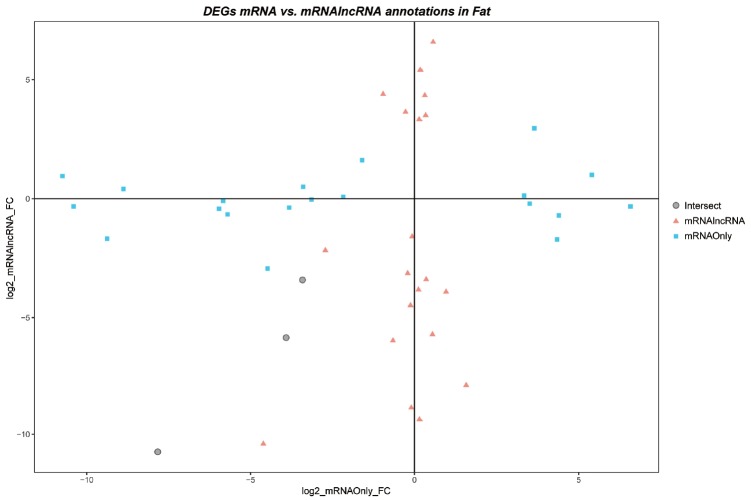
FC-FC plot of adipose tissue. The FC-FC plot of fat indicates that the fat has the least number of intersecting genes (circle with black border), and most pipeline-specific genes (squares and triangles). Adipose tissue is the most susceptible to the addition of lncRNA annotation.

As the significant DEG list changes, we can deduce that there are interactions between conjugate lncRNAs and mRNAs that affect the count of mRNAs; this resulted in a decrease or increase in fold-change of particular genes as shown in (Figure 1). The triangles and squares are the examples of genes that have changed expression patterns due to pipelines. These genes with significant FC in one pipeline is not significantly different in the other. While focusing on the plot in fat, (Figure 3), the proportion of intersection is the lowest among the 4 tissues, which is an indication of tissue specificity of lncRNA and mRNA expression.

In addition, the interpretation in sex biasedness is a clear indicator of the problem in the discordant pipelines. A female up-regulated gene can be changed to a down-regulated gene by the inclusion of lncRNA annotation. Some of the reported sexually-biased genes that were simply defined and detected from previous works may comprise ambiguous genes as shown in our dataset. As an example, the nicotine addiction term is found as a significant KEGG pathway for the mRNA only pipeline, which disappears from the significant table of lncRNA added pipeline. Female down-regulated genes accounted for the pathway in the original pipeline; this could have led to an interpretation that female cattle are less likely to be addicted to nicotine. However, since it disappears in the new pipeline, we can say that the pathway upregulation is ambiguous and requires further validation.

As a first to revisit a public RNA-seq data with a new reference and lncRNA annotation, we have successfully identified a renewed list of sexually dimorphic genes in cattle across four tissues. We believe the intersection genes are more plausible DEG candidates between the two groups, male and female cattle, and such practice of comparing our pipeline (added lncRNA) with the original pipeline provide additional information that is valuable to the bioinformaticians. Moreover, the importance of technical validation, such as qRT-PCR, is imperative for expression analysis since the preprocessing steps—like the reference and annotation— in our study, can alter the statistically significant results. Not to mention the discordance, of DEG list in downstream analyses and interpretation, highlights the problems in the so-called common practices. All in all, we provide a renewed list of candidate genes that may have more direct relation with sexual dimorphism in cattle, and insights to RNA-seq analysts with a new pipeline of checking the DEG list with a second pipeline-run adjusting for genic lncRNA quantification.

## Supplementary materials



Supplementary Figure 1. MDS plot and clusters based on raw expression counts. A contaminated sample, M 002057817341 KY, which is clustered by itself has been removed.

Supplementary Figure 2. Example of a mRNA and lncRNA based gene annotation sharing a loci in the antisense strand, by the authors of [21].

Additional Figures 3. boxplots of top 10 genes in Muscle (mRNA only). The log2 TMM normalized values of male vs. females are illustrated as a boxplot.

Additional Figures 4. boxplots of top 10 genes in Fat (mRNA only). The log2 TMM normalized values of male vs. females are illustrated as a boxplot.

Additional Figures 5. boxplots of top 10 genes in Pituitary Gland (mRNA+lncRNA). The log2 TMM normalized values of male vs. females are illustrated as a boxplot.

Additional Figures 6. boxplots of top 10 genes in Liver (mRNA+lncRNA). The log2 TMM normalized values of male vs. females are illustrated as a boxplot.

Additional Figures 7. boxplots of top 10 genes in Muscle (mRNA+lncRNA). The log2 TMM normalized values of male vs. females are illustrated as a boxplot.

Additional Figures 8. boxplots of top 10 genes in Fat (mRNA+lncRNA). The log2 TMM normalized values of male vs. females are illustrated as a boxplot.

Supplementary Table 1. A combined table of the featureCounts summary file for all samples.

Supplementary Table 2. Full table of concordant (intersect) genes for the 4 respective tissues.

Supplementary File 1. Excerpt from (13) for animal handling procedure.

## Figures and Tables

**Table 1:** The number of detected DEGs in the two protocols

Type1)	Muscle	Adipose tissue	Pituitary gland	Liver
Intersect	17	3	33	23
mRNA only	20	20	7	8
mRNA and lncRNA	21	20	7	8

lncRNA, long non-coding RNA; DEGs, differentially expressed genes.

1) The three types in the table are intersection DEG between the two pipelines and each pipeline-specific DEGs.

**Table 2:** Summary of GO terms for both pipelines in their respective tissues

Tissue	Pipeline1)	Term	Count	p-value	Fold enrichment
P. Gland	mRNA	GO:0016998~cell wall macromolecule catabolic process	5	**3.69E-08**	**135**
P. Gland	mRNA	GO:0050829~defense response to Gram-negative bacterium	5	**1.61E-06**	**55.38462**
P. Gland	mRNA	GO:0019835~cytolysis	4	**9.66E-06**	**90.94737**
P. Gland	mRNA	GO:0050830~defense response to Gram-positive bacterium	5	**1.05E-05**	**34.83871**
P. Gland	mRNA	GO:0007417~central nervous system development	3	**0.004737**	**28.17391**
P. Gland	mRNA	GO:0007155~cell adhesion	4	**0.006694**	**9.988439**
P. Gland	mRNA	GO:0007586~digestion	2	**0.056749**	**33.23077**
P. Gland	mRNA	***GO:0060291~long-term synaptic potentiation***	2	0.058868	32
P. Gland	mRNA	GO:0021766~hippocampus development	2	**0.071487**	**26.18182**
P. Gland	mRNA	GO:0005615~extracellular space	**14**	**1.20E-08**	**7.120197**
P. Gland	mRNA	GO:0005578~proteinaceous extracellular matrix	4	**0.005405**	**10.79679**
P. Gland	mRNA	GO:0003796~lysozyme activity	6	**2.25E-10**	**154.0588**
P. Gland	mRNA	GO:0005540~hyaluronic acid binding	2	**0.045525**	**41.57143**
P. Gland	mRNA	GO:0005201~extracellular matrix structural constituent	2	**0.07886**	**23.59459**
Adipose	mRNA	***GO:0003774~motor activity***	2	0.029846	59.52273
Muscle	mRNA	***GO:0009952~anterior/posterior pattern specification***	2	0.048822	36.42688
P. Gland	mRNA+lncRNA	GO:0016998~cell wall macromolecule catabolic process	5	2.79E-08	144
P. Gland	mRNA+lncRNA	GO:0050829~defense response to Gram-negative bacterium	5	1.22E-06	59.07692
P. Gland	mRNA+lncRNA	GO:0019835~cytolysis	4	7.86E-06	97.01053
P. Gland	mRNA+lncRNA	GO:0050830~defense response to Gram-positive bacterium	5	8.01E-06	37.16129
P. Gland	mRNA+lncRNA	GO:0007417~central nervous system development	3	0.004154	30.05217
P. Gland	mRNA+lncRNA	GO:0007155~cell adhesion	4	0.005542	10.65434
P. Gland	mRNA+lncRNA	GO:0007586~digestion	2	0.053183	35.44615
P. Gland	mRNA+lncRNA	GO:0021766~hippocampus development	2	0.067028	27.92727
P. Gland	mRNA+lncRNA	GO:0005615~extracellular space	12	1.21E-06	6.103026
P. Gland	mRNA+lncRNA	GO:0005578~proteinaceous extracellular matrix	4	0.005405	10.79679
P. Gland	mRNA+lncRNA	GO:0003796~lysozyme activity	6	2.25E-10	154.0588
P. Gland	mRNA+lncRNA	***GO:0008236~serine-type peptidase activity***	2	0.037011	51.35294
P. Gland	mRNA+lncRNA	GO:0005540~hyaluronic acid binding	2	0.045525	41.57143
P. Gland	mRNA+lncRNA	GO:0005201~extracellular matrix structural constituent	2	0.07886	23.59459
Adipose	mRNA+lncRNA	***GO:0005737~cytoplasm***	6	0.020001	2.983789
Muscle	mRNA+lncRNA	***GO:0008203~cholesterol metabolic process***	2	0.059757	31.27602
Muscle	mRNA+lncRNA	***GO:0007059~chromosome segregation***	2	0.080017	23.11706

GO, gene ontology; lncRNA, long non-coding RNA.

1) The two pipelines are separated by a double-line break. The pipeline specific terms and their specific values are bolded if there are differences between the two pipelines’ result.

**Table 3:** Summary of KEGG pathways for both pipelines in their respective tissues

Tissue	Pipeline1)	Term	Count	p-value	Fold enrichment
P. Gland	Mrna	bta04970:Salivary secretion	3	**0.006106**	**22.84036**
P. Gland	mRNA	***bta05033:Nicotine addiction***	2	0.059304	30.09127
Liver	mRNA	bta00140:Steroid hormone biosynthesis	**3**	**0.002403**	**36.2823**
Liver	mRNA	***bta00830:Retinol metabolism***	2	0.072718	24.1882
Liver	mRNA	***bta05204:Chemical carcinogenesis***	2	0.08862	19.6961
Muscle	mRNA	***bta04972:Pancreatic secretion***	2	0.049696	31.59583
P. Gland	mRNA+lncRNA	bta04970:Salivary secretion	3	**0.008538**	**19.57745**
Liver	mRNA+lncRNA	bta00140:Steroid hormone biosynthesis	**2**	**0.058602**	**29.56335**

KEGG, Kyoto encyclopedia of genes and genomes; lncRNA, long non-coding RNA.

1) The two pipelines are separated by a double-line break. The pipeline specific terms and their specific values are bolded if there are differences between the two pipelines’ result.
