# Supplementary material for: Multi-tissue observation of the long non-coding RNA effects on sexually biased gene expression in cattle
Source: Asian-Australas J Anim Sci. 2018 Nov 28;32(7):1044–51. doi: 10.5713/ajas.18.0516 (PMC6603329; doi:10.5713/ajas.18.0516)
Supplement: Supplementary file 1 [file ajas-18-0516-suppl.pdf]

Supplementary materials

Supplementary figure legends

Supplementary Figure 1. MDS plot and clusters based on raw expression counts. A contaminated sample, M 002057817341 KY, which is clustered by itself has been removed.

Supplementary Figure 2. Example of a mRNA and lncRNA based gene annotation sharing a loci in the antisense strand, by the authors of [21].

The following additional figures provide extra information of the genes detected in each method/tissue. They hold minor importance in the article itself:

Additional Figures 1.boxplots of top 10 genes in Pituitary Gland (mRNA only). The log2 TMM normalized values of male vs. females are illustrated as a boxplot.

Additional Figures 2. boxplots of top 10 genes in Liver (mRNA only). The log2 TMM normalized values of male vs. females are illustrated as a boxplot.

Additional Figures 3. boxplots of top 10 genes in Muscle (mRNA only). The log2 TMM normalized values of male vs. females are illustrated as a boxplot.

Additional Figures 4. boxplots of top 10 genes in Fat (mRNA only). The log2 TMM normalized values of male vs. females are illustrated as a boxplot.

Additional Figures 5. boxplots of top 10 genes in Pituitary Gland (mRNA+lncRNA). The log2 TMM normalized values of male vs. females are illustrated as a boxplot.

Additional Figures 6. boxplots of top 10 genes in Liver (mRNA+lncRNA). The log2 TMM normalized values of male vs. females are illustrated as a boxplot.

Additional Figures 7. boxplots of top 10 genes in Muscle (mRNA+lncRNA). The log2 TMM normalized values of male vs. females are illustrated as a boxplot.

Additional Figures 8. boxplots of top 10 genes in Fat (mRNA+lncRNA). The log2 TMM normalized values of male vs. females are illustrated as a boxplot.

Supplementary Table legends

Supplementary Table 1. A combined table of the featureCounts summary file for all samples.

Supplementary Table 2. Full table of concordant (intersect) genes for the 4 respective tissues.

Supplementary Files

Supplementary File 1. Excerpt from (13) for animal handling procedure.

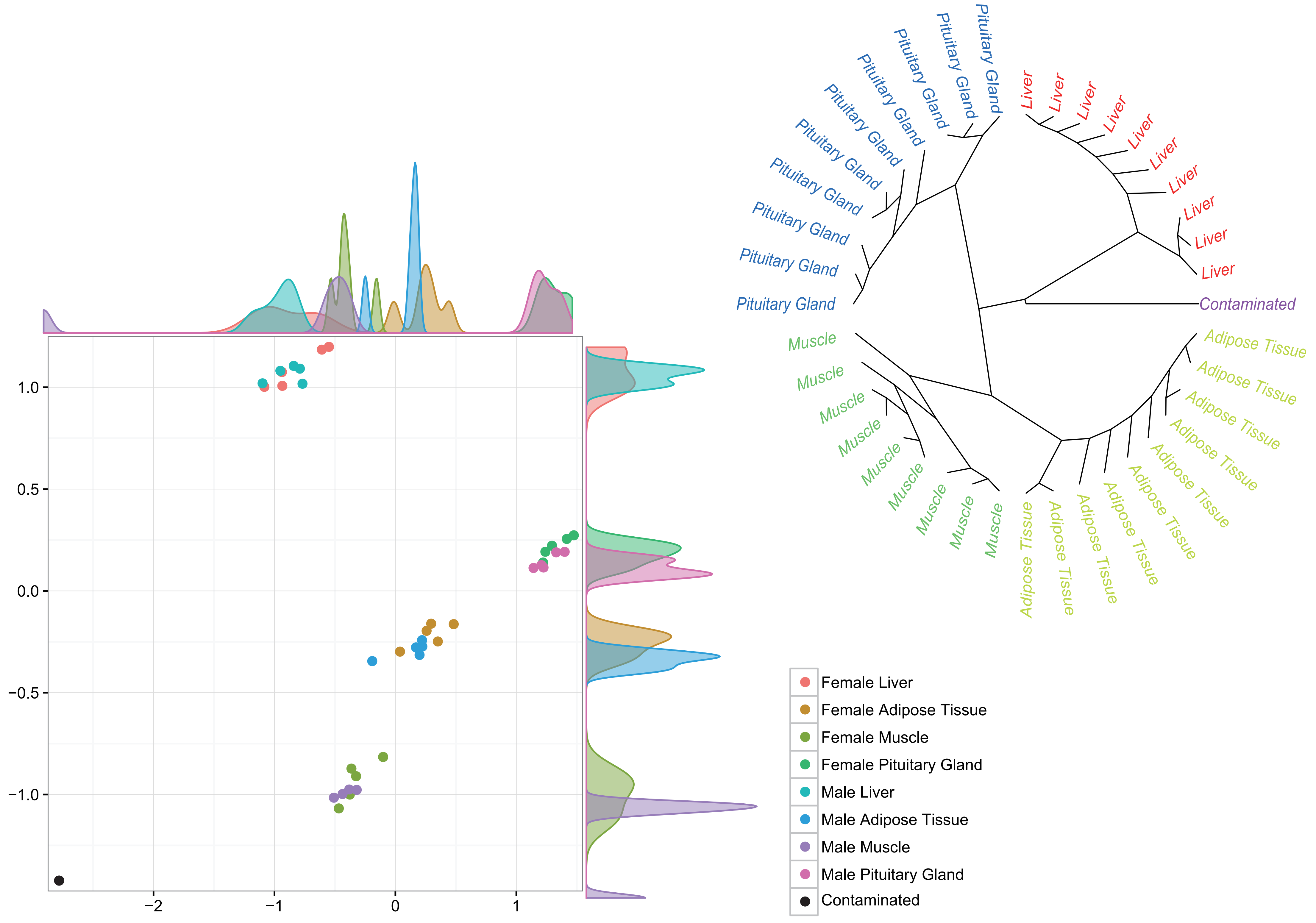
Pituitary Gland
Pituitary Gland
Pituitary Gland
Pituitary Gland
Pituitary Gland
Pituitary Gland
Pituitary Gland
Pituitary Gland
Pituitary Gland
Pituitary Gland
Liver
Liver
Liver
Liver
Liver
Liver
Liver
Liver
Liver
Liver
Contaminated
Muscle
Muscle
Muscle
Muscle
Muscle
Muscle
Muscle
Muscle
Muscle
Adipose Tissue
Adipose Tissue
Adipose Tissue
Adipose Tissue
Adipose Tissue
Adipose Tissue
Adipose Tissue
Adipose Tissue
Adipose Tissue
Adipose Tissue
1.0
0.5
0.0
−0.5
−1.0
−2
−1
0
1
Female Liver
Female Adipose Tissue
Female Muscle
Female Pituitary Gland
Male Liver
Male Adipose Tissue
Male Muscle
Male Pituitary Gland
Contaminated
