# Supplementary material for: Multi-tissue observation of the long non-coding RNA effects on sexually biased gene expression in cattle
Source: Asian-Australas J Anim Sci. 2018 Nov 28;32(7):1044–51. doi: 10.5713/ajas.18.0516 (PMC6603329; doi:10.5713/ajas.18.0516)
Supplement: Supplementary file 12 [file ajas-18-0516-suppl11.docx]

**Animal handling and RNA-seq procedures**

Excerpt from ([Seo, Caetano-Anolles et al. 2016](#_ENREF_15)) on Animal handling and RNA-seq procedures as we used the published dataset and refined the study. [These are direct quotes from the article, and should be removed from the manuscript while checking for plagiarism.]

“All animal procedures were approved by the National Institute of Animal Science Institutional Animal Use and Care Committee (NIASIAUCC), Republic of Korea, and performed in accordance with the animal experimental guidelines provided by NIASIAUCC. Samples were collected from Korean cattle raised in the Daekwanryung experimental branches of the National Institute of Animal Science (NIAS). 10 cattle were slaughtered at age of (>22 months) and carcass weight was 353 ± 36 kg after slaughter. Abdominal adipose tissue, liver, intact longissimus dorsi muscle, and pituitary gland tissue samples were immediately separated after slaughter. Tissue samples were stored at -80 °C, and total RNA was isolated from the four tissues using the TRIzol reagent (Invitrogen) based on the manufacturer instructions. Total RNA quality and quantity was verified using a NanoDrop1000 spectrophotometer (Thermo Scientific, Wilmington, DE, USA) and Bioanalyzer 2100 (Agilent technologies,Palo Alto CA, USA). The mRNA in total RNA was converted into a library of template molecules suitable for subsequent cluster generation using the reagents provided in the Illumina ® TruSeq™ RNA Sample Preparation Kit. In summary, mRNA was purified using poly-A selection, then chemically fragmented and converted into single-stranded cDNA using random hexamer priming. The second strand is then generated to create double-stranded cDNA that is ready for TruSeq library construction. The short ds-cDNA fragments were then connected with sequencing adapters, and suitable fragments were separated by agarose gel electrophoresis. Finally, truseq RNA libraries were built by PCR amplification, quantified using qPCR according to the qPCR Quantification Protocol Guide, qualified using the Agilent Technologies 2100 Bioanalyzer. (Agilent technologies,Palo Alto CA, USA). Based on the generated RNA libraries, paired-end sequencing (101 bp read-length and approximately 150 to 180 insert size) was performed using the HiSeq™ 2000 platform (Illumina,San Diego, USA). Next, to measure transcriptome levels with generated RNA-seq reads we performed the following widely used RNA-seq pipeline: (1) We employed Trimmomatic (v0.32) ([Bolger, Lohse et al. 2014](#_ENREF_1)) with following option: PE -phred33 ILLUMINACLIP:TruSeq3-PE.fa:2:30:10 MINLEN:75 2 for making clean reads.”
